# Honeycomb Map: A Bioinspired Topological Map for Indoor Search and Rescue Unmanned Aerial Vehicles

**DOI:** 10.3390/s20030907

**Published:** 2020-02-08

**Authors:** Ricardo da Rosa, Marco Aurelio Wehrmeister, Thadeu Brito, José Luís Lima, Ana Isabel Pinheiro Nunes Pereira

**Affiliations:** 1 Federal Institute of Education, Science and Technology—Parana (IFPR), 85814-800 Campus Cascavel, Brazil; 2 Campus Curitiba, Federal University of Technology—Parana (UTFPR), 80230-901 Curitiba, Brazil; wehrmeister@utfpr.edu.br; 3 Campus de Santa Apolónia, Instituto Politécnico de Bragança (IPB), Research Centre in Digitalization and Intelligent Robotics (CeDRI), 5300-253 Bragança, Portugal; brito@ipb.pt (T.B.); jllima@ipb.pt (J.L.L.); apereira@ipb.pt (A.I.P.N.P.); 4 INESC TEC - INESC Technology and Science, 4200-465 Porto, Portugal

**Keywords:** multi-robot, UAV, bioinspired map, topologic mapping, map exploration

## Abstract

The use of robots to map disaster-stricken environments can prevent rescuers from being harmed when exploring an unknown space. In addition, mapping a multi-robot environment can help these teams plan their actions with prior knowledge. The present work proposes the use of multiple unmanned aerial vehicles (UAVs) in the construction of a topological map inspired by the way that bees build their hives. A UAV can map a honeycomb only if it is adjacent to a known one. Different metrics to choose the honeycomb to be explored were applied. At the same time, as UAVs scan honeycomb adjacencies, RGB-D and thermal sensors capture other data types, and then generate a 3D view of the space and images of spaces where there may be fire spots, respectively. Simulations in different environments showed that the choice of metric and variation in the number of UAVs influence the number of performed displacements in the environment, consequently affecting exploration time and energy use.

## 1. Introduction

Mobile robotics is being applied more often to not only solve problems found in industrial environments, but also applied to services and home uses. For example, robots can be used in the process of warehouse automation, space monitoring, and house cleaning. These new applications show that a mobile robot can perform complex tasks while navigating unknown environments and avoiding unexpected obstacles by reacting to environmental stimuli [1]. Another application of mobile robotics is in the support of rescue teams in natural-disaster or catastrophe situations. Exploration might put the life of rescue-team professionals in danger. The use of Unmanned Aerial Vehicles (UAV) may assist rescue activities, especially in indoor areas where the arrival or movement of a ground robot is sometimes impossible. Access to unknown indoor areas requires techniques for defining the space where a robot is positioned, generating environmental mappings in order to aid teams in the reconnaissance of these areas where the use of global positioning systems (GPS) is unavailable. Thus, an autonomous robot must deal with two critical problems to survive and navigate in its environment: mapping the environment, and searching for its own location in the map [2].

For rescue environments, the time for space recognition becomes critical. Thus, the use of multiple robots can reduce environment exploration time. The collective construction of a map that is used to displace both multiple robots and the rescue team must represent spaces where it is possible to move and points that need more attention, such as human-temperature recognition, toxic elements, fires, and other factors that could be life-threatening.

This work proposes a mapping approach that was bioinspired by honeycomb construction. Honeybees use hexagonal-pattern cylinders to progressively build a complex structure by adding wax produced and manipulated by several bees [3]. This hexagonal structure allows the construction of combs with less wax (material saving), with the capacity for more storage. The construction of a honeycomb structure starts from a cell floor. Then, the structure is progressively extended in depth by adding more materials around the cell walls. The hive combs are the result of the collective work of hundreds of bees. There is no central commander/master for the building process. The individuals follow simple rules related to environmental construction (e.g., only one bee at a time can build a particular comb, and a new cell must be adjacent to an existing cell), so that this environment influences behavior, which, in turn, transforms the environment, it being a mechanism of synergy [3].

The scope of this work is in the application of simulated models of UAVs with similar configuration, and in addition, it will make use of simulation environments to validate the developed method. In this way, details and restrictions of communication technologies are abstracted.

## 2. Related Works

### 2.1. Map Generation

Building an environment map is necessary for both robot exploration and in simultaneous localization and mapping (SLAM) tasks. In [4], map generation was partitioned into three parts: metric, topological, and hybrid maps. Cartographic maps are able to make use of Vector map ([5,6,7]); however, they are not the focus of this work.

#### 2.1.1. Metric Maps

Metric maps try to extract the features and geometric properties of the environment, and they are represented as a grid, geometric, or feature map [8]. Often, metric maps are probabilistic [4], and establish methods for modeling noise and its effects on environmental modeling. The approaches are based on a Bayesian filter, graph-based SLAM, and submap-joining SLAM.

#### 2.1.2. Topological Maps

Topological maps represent the environment in graphs, where nodes represent places and objects of interest, and edges represent the spatial relationship or path between nodes [4]. In addition to providing a more compact representation of the environment than metric maps, topological maps provide a higher-level symbolic understanding for planning and navigation tasks. While metric maps are achieved with odometry-error accumulation, topological maps are built without the worry of metric aspects. Odometry errors that are accumulated between graph nodes do not necessarily accumulate through the global map.

#### 2.1.3. Hybrid Maps

Hybrid maps combine the advantages of metric and topological mapping. Topological mapping is applied for a global view of the environment, while metric mapping is applied to smaller areas, which reduces computational complexity during metric-information processing. A hybrid-map form is the use of each topological-map node to represent a small metric map, and edges between nodes represent the path from the center point of one metric map to the center point of the next metric map [4].

### 2.2. Multiple Robots in Environment Mapping

Solutions that use multiple robots are characterized by the application of homogeneous and heterogeneous robots. Many related works make use of SLAM algorithms, but the focus of this work is environment exploration. Thus, works that make use of SLAM were considered for understanding the way they build the maps.

In [9], the authors performed collaborative space mapping with UAV and Unmanned Ground Vehicle (UGV) modeling through complementary maps. While the UGV does 2D area mapping, the UAV does 3D mapping of orthogonal objects in the environment. In [10], the authors presented a practical application, which is the mapping of areas struck by earthquakes. This being an implementation that uses a UAV and UGV, operation is semiautonomous. That happens because the UGV is remotely controlled, but when it faces obstacles it cannot overcome, the UAV autonomously does the mapping of the area. The execution of a 3D SLAM is done by the UAV via an RGB-D sensor, and by the UGV with a laser scanner. In [11], the UAV implements a Parallel Tracking and Mapping (PTAM)on the basis of sonar readings, while the UGV executes a Visual SLAM (VSLAM) fed by RGB-D and laser sensors. The work’s goal was heterogeneous exploration using integer programming. The UGV has its own VSLAM and, for places that it cannot explore, the UAV is put in action using PTAM. UAV data via PTAM are then sent to the UGV and integrated in a VSLAM.

Some works that only use UAVs are presented: [12] uses a swarm to distribute areas to be explored by the UAVs. The focus is the use of UAVs for both hunting and cleaning. Here, in a group of many UAVs, one is defined as a sentinel and partitions the area for exploration. The work of [13] modified the PTAM algorithm for multiple agents using monocular cameras. Environment exploration is done cooperatively with recognition of points of interest. The definition of exploration is done via auction, where each bid is the linear distance of each UAV to the point being explored. The shortest distance wins the auction. In [14], an adaptation of PTAM (Parallel Tracking and Multiple Mapping—PTAMM) with the use of RGB-D, inertial measurement unit (IMU), and infrared (IR) sensors was presented. The work did localization and mapping using RGB-D sensors. A characteristic of this work is that it decomposed a 3D SLAM problem in a monocular SLAM with sparse representation.

There are solutions that implemented cooperative indoor mapping by using only UGVs [15,16,17,18,19]. In [15], heterogeneous robots were used in 2D and 3D area mapping using laser scanners, performing 3D and 2D cooperative mapping via autonomous agent navigation. Here, each robot builds a local map and sends the relevant data to a central server, where the data are joined with existing data using join-compatibility branch and bound (JCBB) implementation. In [16], the authors adapted the FastSLAM algorithm for multiple agents by also using laser scanners. Presenting a version of FastSLAM adapted to multiple UGV robots, it could perform cooperative mapping with the stigmergic potential field (SPF) technique, which represents behavioral influences of gathered data from the operational environment of one of the agents. In [17], the UGVs executed a VSLAM via a monocular camera. The creation of cooperative SLAM was based on salient landmarks to represent prominent characteristics. For that, each robot performs its own monocular SLAM with Extended Kalman Filter (EKF). The merge algorithm uses duplicated landmarks to increase the accuracy of the centralized map. In [18], a laser and webcam were used to model an area. By employing multiple autonomous UGVs, this work performs exploration with teams of robots for learning. Each robot creates a partial 3D map that it shares with other robots in its communication range. A global map is created on the basis of matching poses and mutual characteristics found in individual maps. The authors in [19] presented an implementation of multiple GraphSLAM using a stereo camera. Here, autonomous UGVs perform 6D mapping of an area using graph topology to separate uncertainty estimates of the local filters of multiple robots in a SLAM graph.

## 3. Methodology—Bioinspired Mapping Method

For [20], an exploration task is the combination of both mapping and robot motion-control activity.

This work proposes an environment exploration method with multiple UAVs inspired by how bees build hives. The authors in [3] discussed how bees perform hive construction. Following the behavior of bees in the construction of each honeycomb, UAVs perform the build and exploration map in a similar way, where combs are represented as hexagons. Each honeycomb can have only one bee occupying its space, so each hexagon can hold a maximum of one UAV. The built map is a collection of hexagons.

**Figure 1:**
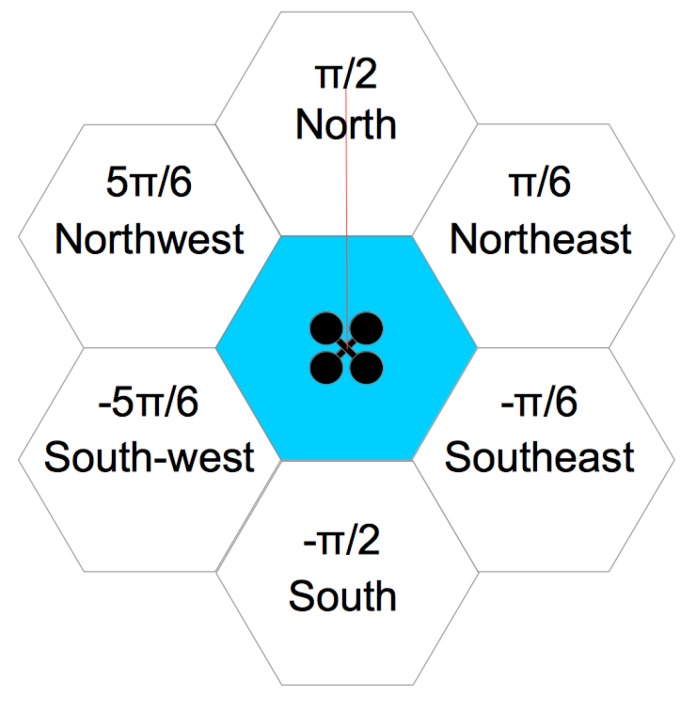
Unmanned Aerial Vehicle (UAV) in hexagon exploration.

The construction of a beehive begins with the work of the first bee, which begins construction of the first honeycomb using wax to build its walls. Similarly, in the proposed method, a first UAV, identified as the sentinel, generates the first map hexagon, checking whether there are adjacencies for each of the six sides (honeycomb walls). In this case, the term adjacency means the possibility for a UAV to move from one hexagon to another. Thus, a hexagon exists on the map if and only if it is possible for a UAV to fully access it from another hexagon on at least one of its six sides, so obstacles cannot exist between the center of one hexagon and the center of the other hexagon. Figure 1 shows a UAV exploring a hex that should rotate at six angles: π/2,π/6,-π/6,-π/2,-5π/6, and 5π/6. Each evaluated hexagon with possible adjacency is marked with an identifier.

**Figure 2:**
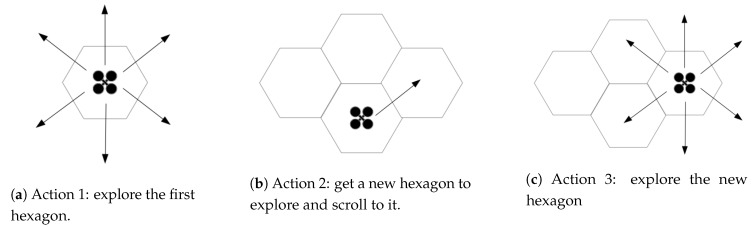
Exploration stages.

Briefly, the UAV explores the hexagon in each of its six angles, sets a new hexagon to explore and moves to this, starts a new exploration. The Figure 2 shows this action.

Once the sentinel UAV finishes the first scan, all UAVs can start searching for spaces to explore. To control the hexagons identified in the reading process from each of the six angles, some structures are used. To record the identifiers (ids) of the explored hexagons, a list called “visited hexagon list” is used. When the UAV rotates and finds adjacency for a new hexagon, a new id is generated and added into a structure called a “not visited hexagon list”. Thus, a UAV searching for a hexagon to explore should perform this search in the “not visited hexagon list”.

**Figure 3:**
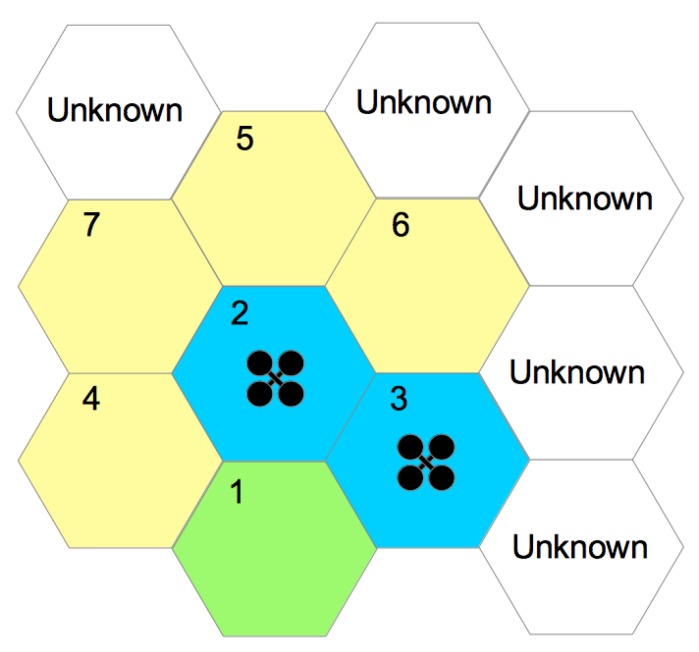
Multiple UAV exploration: green hexagon, explored place; yellow hexagons, places that have adjacency, but not yet explored; blue hexagons, places that are explored by UAV; white hexagons, unknown places that are mapped in future steps.

At the end of hexagon exploitation, id is removed from the latter list. Figure 3 presents two UAVs exploring a given space. In this case, exploration started with hexagon 1, which was already explored. For illustration purposes, hexagon 1 is green, indicating that it was already fully explored. In its exploration process, adjacencies were identified with hexagons 2–4, which were inserted into the “not visited hexagon list”. When a UAV began exploring hexagon 2, hexagons 5–7 were identified. Blue hexagons represent spaces in exploration, while yellow ones are those that were identified but not yet explored. The exploration process ends when the “not visited hexagon list” is empty.

### 3.1. Environment Exploration

**Figure 4:**
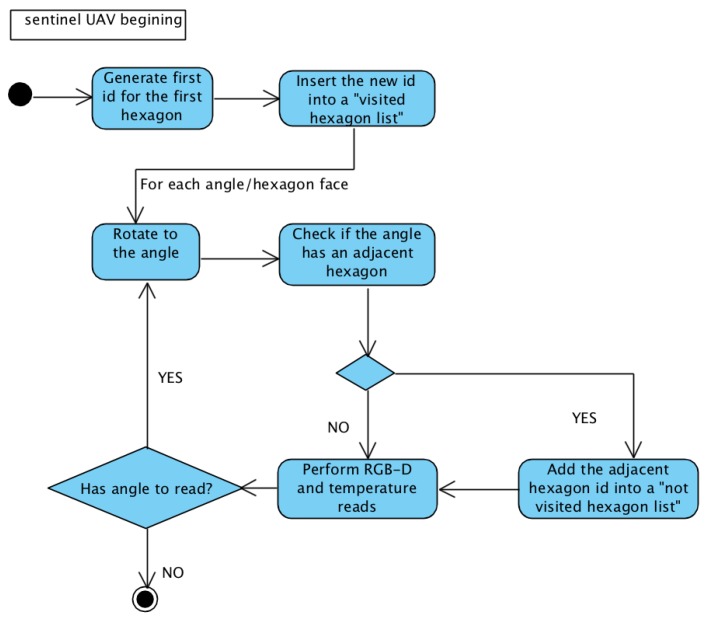
First sentinel UAV exploration.

**Figure 5:**
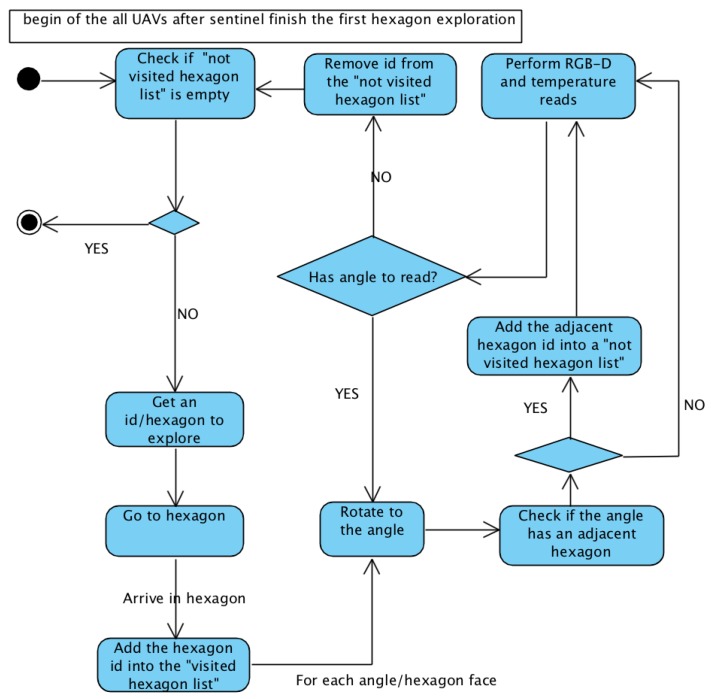
All UAVs after first sentinel exploration.

Figure 4 and Figure 5 present state diagrams of the scanning activity of both sentinel and other UAVs. The sentinel UAV only behaves differently in the first exploration (where it generates the first *id* from point xyz from its placement); in the others, it has the default behavior of the other UAVs.

#### 3.1.1. Checking If *“Not Visited Hexagon List”* Is Empty

This is the stopping criterion of the exploring algorithm. Each uncovered discovered hexagon is inserted into the “not visited hexagon list”. When a UAV receives a id to explore, it remains in the “not visited hexagon list” to the end of the exploration, but the UAVs that it exploits are registered. This ensures that no UAV stops the exploration process without actually having any new spaces to explore. For example, at one point in the exploration, one UAV may have finished its exploration, while another is working. If no unvisited hexagons are currently available, the first UAV waits for possible discoveries of the second UAV, which is still in exploration activity. If no new hexagon is discovered, then exploration is finished, or new explorations process are done again.

#### 3.1.2. Getting Id/Hexagon to Explore

When a UAV is free (no hexagons in the exploration process), it seeks a new place to explore, which is done in the “not visited hexagon list”. To define which is assigned to the UAV, two metrics were defined for different simulations: First-In–First-Out (FIFO) and Euclidean distance. The FIFO metric assigns to the UAV that unvisited hexagon than has been awaiting exploration for the longest, so the first discoveries are the first to be explored. The second metric defines that the hexagon to be explored by the UAV is the one with the smallest Euclidean distance from the initial hexagon (id 1).

#### 3.1.3. Go to Hexagon

Once the UAV gets a hexagon to explore, it must travel there. The UAV only transitions through familiar and accessible spaces. Thus, given the hexagon where the UAV is located and the target, one path is defined to go. This path is built from Dijkstra’s algorithm [21], with an adapted version from [22]. With this path, the UAV travels the map until it reaches its target.

#### 3.1.4. Add Hexagon Id into “Visited Hexagon List”

When the UAV finishes moving along the path defined by Dijkstra’s algorithm, it is in the hexagon to explore. At the beginning of the exploration activity, hexagon id is inserted into the “visited hexagon list”. This ensures that a UAV identifies a hexagon already found and identified by another UAV, not creating a new id for the same space.

#### 3.1.5. Rotate to Angle and Check If Angle Has Adjacent Hexagon

**Figure 6:**
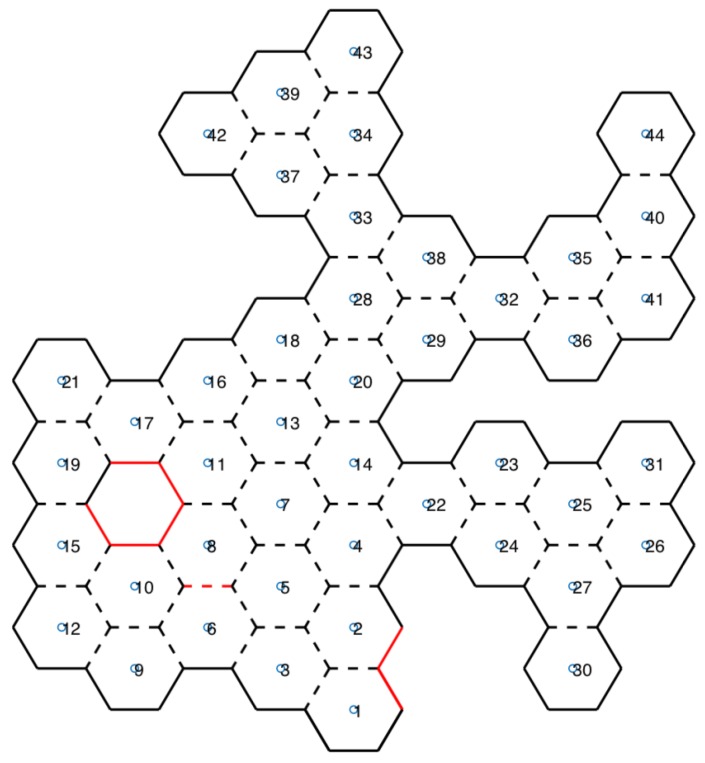
Honeycomb map.

For each six sides of the hexagon (six angles), the UAV should rotate and check for adjacency: a sensor checks if it is possible for the UAV to access the center of the neighboring hexagon; in other words, if there are no obstacles between the two hexagons. If so, adjacency is added to an adjacency matrix. Each angle of the explored hexagon is identified in the honeycomb map with dashed lines if there is adjacency at that angle, or with continuous lines if there is none, as shown in Figure 6.

#### 3.1.6. Add Adjacent Hexagon Id into “Not Visited Hexagon List”

When the sensor reading discovers an adjacent hexagon for each of the six angles, and it is not in the “visited hexagon list”, it sends it to the “not visited hexagon list”, if it is not already there (this is a newly discovered hexagon).

#### 3.1.7. Perform RFB-D and Temperature Reads

Map information is available for both UAVs to control their movements in the environment, and for rescue teams to know the space that can be navigated. In addition to obstacle sensors, RGB-D and temperature sensors are used. RGB-D sensors read the 3D angle of the UAV, and a cube view is then built to aid rescue teams in space recognition. At the same time, a thermal sensor reads the temperature from the same angle. If a temperature higher than a reference value is found, it is identified and a photo of the location is taken. In honeycomb map, this scenario is represented with red lines, as shown in Figure 6.

The RGB-D reading returns a matrix structure. The matrix size (nxm) and the range of RGB-D sensor are set in a V-REP simulator in the sensor settings. Matrix values are between 0 and 1, where 0 is very close to and 1 very far from the sensor. When the UAV reads RGB-D, the 3D data, and the angle and position of the UAV during the reading are recorded. These data are transformed from a perspective to a global point. For instance, let *R* be the cube size, amplitudeRGBD the extent of the RGB-D sensor, buffer the RGB-D matrix read, xn and yn the buffer dimension, angUAV the UAV Euler angle, and posUAV the xyz UAV position. Algorithm 1 brings the data transformation.
**Algorithm 1**: RGB-D transformation algorithm.1**function** [ xc , yc , zc ] = TransformRGBD(R, amplitudeRGBD,2             buffer , xn , yn , angUAV, posUAV)3xc = 0 ;4yc = 0 ;5zc = 0 ;6deltaAngleRGBD = double ( amplitudeRGBD)/double ( xn ) ;7**for** i =1:yn8  **for** j =1: xn9   **if** ( double ( buffer ( i , j ) ) <0.99)10    angUAVz=rad2deg (angUAV(3) ) ;11    —nz is the distance in meter.12    —RGB-D is **set** to 2m13    nz=double ( buffer ( i , j ) ) * 2 ;14    **if** ( j ==1)15     dtAng=0;16    **elseif** ( j ==xn )17     dtAng=amplitudeRGBD ;18    **else**19     dtAng=deltaAngleRGBD*double ( j ) ;20    **end**21    **if** ( dtAng < amplitudeRGBD/2)22     alfa =double (angUAVz) +( ( amplitudeRGBD/2)−dtAng ) ;23    **else**24     alfa =double (angUAVz)−(dtAng−(amplitudeRGBD/2) ) ;25    **end**26    alfarad=deg2rad ( alfa ) ;27    —Sine’ s Law28    dy = double (nz * **sin** ( double ( alfarad ) )/**sin** ( deg2rad ( 9 0 ) ) ) ;29    dx = nz * **sin** ( double ( deg2rad(180−90−alfa ) ) )/**sin** ( deg2rad ( 9 0 ) ) ;30    —calculate dz31    angUAVx = rad2deg (angUAV( 1 ) ) ;32    **if** ( i ==1)33     dtAngz=0;34    **elseif** ( i==xn )35     dtAngz=amplitudeRGBD ;36    **else**37     dtAngz = deltaAngleRGBD*double ( i ) ;38    **end**39    **if** ( dtAngz < amplitudeRGBD/2)40     alfaz=double (angUAVx) +( ( amplitudeRGBD/2)−dtAngz ) ;41    **else**42     alfaz=double (angUAVx)−(dtAngz−(amplitudeRGBD/2) ) ;43    **end**44    dz = nz * **sin** ( double ( deg2rad ( alfaz ) ) )/**sin** ( deg2rad ( 9 0 ) ) ;45    xp = posUAV( 1 ) + dx ;46    yp = posUAV( 2 ) + dy ;47    zp = posUAV( 3 ) + dz ;48    —Discretizing values.49    xc = **round** ( xp/R) * R;50    yc = **round** ( yp/R) * R;51    zc = **round** ( zp/R) * R;52   **end**53  **end**54**end**55**end**

#### 3.1.8. Remove Id from “Not Visited Hexagon List”

At the end of the reading of the six sides of the hexagon, id is removed from the “not visited hexagon list”. Then, the UAV can begin the search for a hexagon to explore again if the “not visited hexagon list” is not empty; otherwise, the UAV’s exploration activity is finished.

### 3.2. Lock Path Resolution

Throughout the exploration process, the various UAVs will be moving towards their targets, and consequently, their paths may cross. Avoiding collisions is a critical point for an environment with multiple robots. Several approaches have been presented, where means of prevention are proposed by optimized programming [23,24,25], potential fields [26], sampling-based methods [27], and others. In general, two concepts are applied [28]: one where robots are free and can change their paths, and another where robots have a fixed path with no possibility of changes. Thus, in the first concept, the focus is on changing paths, while in the second, the focus is on controlling movement and time. In [28] a method for treating deadlock for multiple robots where the path is fixed is discussed. To improve performance, some stopping policies are proposed. With these policies, each robot makes the decision to change or wait for another one. A correct-by-construction synthesis approach to multi-robot mission planning that guarantees collision avoidance with respect to moving obstacles are approach in [29], where has done an integration of a high-level mission planner with a local planner that guarantees collision-free motion in three-dimensional workspaces, when faced with both static and dynamic obstacles.

To avoid collisions, the proposed architecture defines that only one UAV can occupy one hexagon (honeycomb) at a time. Thus, it is necessary to have a record of the hexagon that the UAV currently occupies. To do this, each time a UAV moves from one hexagon to another, it records both which one it is in and what is the next move. Collision is avoided in this way; however, deadlock states can happen.

In the proposed approach, it is assumed that a UAV can be found in three possible states: “in exploration”, “in displacement” or “stopped”. The state “in exploration” means that the UAV is reading the six angles of the hexagon (honeycomb), and generating the mapping data. “In displacement” means that the UAV is moving to a hexagon and make their exploitation. The “stopped” state means that the UAV has no allocated exploration, and is not moving to any honeycomb.

A key element for resolving path blocks in this approach is the Adjacent Degree (AD), which is the number of adjacent hexagons, directly or indirectly, to which the UAV can travel, in order to free the paths. To obtain the AD, each UAV checks how many hexagons are directly adjacent to it, excluding those in which they are occupied by other UAVs. If the AD value is greater than 1, it means that there is space to perform a maneuver to release the passage. If the AD value is 1, the AD value for this adjacent single is searched. The AD value found for the adjacent one will be its value as well.

**Figure 7:**
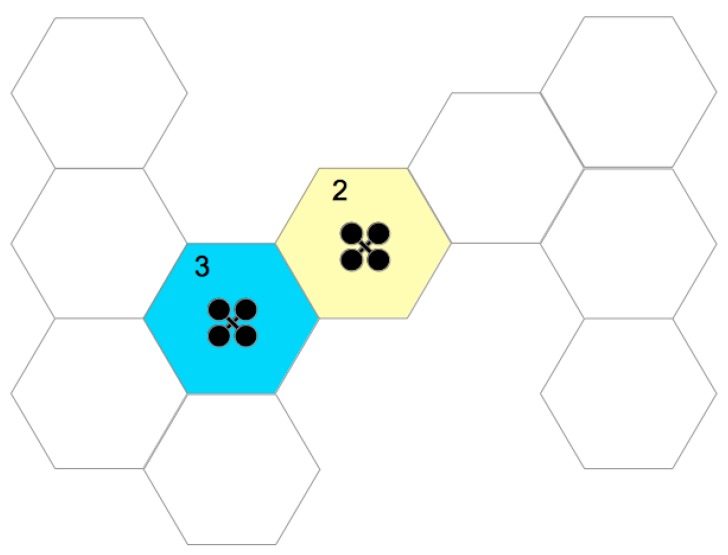
Adjacent Degree: blue hexagon has AD = 3, while yellow hexagon AD = 2.

A comparison between the AD values of each UAV is made, and if there is a conflict between UAVs, the one with the highest AD must give way to the one with the smallest, moving to one of its adjacent hexagons to resolve the deadlock. When he finishes moving, he retraces his trajectory for his hexagon to explore and returns to his tasks. The Figure 7 presents a scenario with two UAVs, where the UAV in the blue hexagon has three adjacent hexagons directly and its AD value is 3, while the one in yellow has a single adjacent one; however, this, in turn, it has two adjacent hexagons, making the UAV AD in the yellow hexagon to be 2.

Considering the existence of several UAVs in the environment, each one identified as A,B,C,...,Z, and A→B representing the UAV who wants to move to the hexagon which is the UAV B. Some cases of path blocking can happen:

- **Case 1—A→B and B→A:**
  In this scenario, UAV A wants to move to the hexagon of UAV B, and at the same time, UAV B wants to move to the hexagon where UAV A. Here, each UAV calculates its adjacent degree (AD). The UAV that has the largest AD will open the way to the other UAV.
- **Case 2—A→B:**
  In this scenario, only UAV A shows that it wants to move to the hexagon of UAV B; however, B will not go to the hexagon of A is. In this case, UAV B may be in an “in exploration” or “stopped” state. If it is in an “in exploration” state, UAV A will recalculate a new path trying to deflect the hexagon occupied by B. If there is only one path, UAV A waits for UAV B to complete its exploration. On the other hand, if UAV B is in a “stopped” state, UAV B itself will identify that UAV A wants to go to the hexagon it occupies. That way, it will calculate your AD and compare it with the UAV A. If your AD is greater, it will move to a free adjacent hexagon, and otherwise, it will try to move to a hexagon adjacent to the UAV A, which causes them to find themselves in Case 1.
- **Case 3—A→B, B→C and C→A:**
  In this case, two UAVs are unable to mutually identify a deadlock. So, it is necessary to check if there is a cyclically blocking. Thus, from the hexagon to which you want to move, UAV A checks if there are any others that want to move to where it is. If this block is detected, the UAV calculates its AD, and if it is greater than 0, it will give space for the resolution of the deadlock. After that, the path to the defined hexagon will be recalculated, and then continue your task.

### 3.3. Simulation

To validate the proposed method, simulations were performed with different scenarios and UAV numbers. Through the simulation, it was possible to verify the proposed approach, i.e., to plan the UAV tasks to map a catastrophic environment. Simulations were created with the following setup: CPU, Intel Xeon with 3.33 GHz 6 Core, 6 GB 1333 MHz DDR3 memory, and GPU ATI Radeon HD 5770 1024 MB.

There are several robot simulation environments, such as Open HRP [30], Gazebo [31], Webots [32] and Virtual Robot Experimentation Platform (V-REP) [33]. In this work, we chose V-REP, which has application programming interfaces (API) that allow communication with many programming languages. The proposed approach was implemented in MATLAB [34].

**Figure 8:**
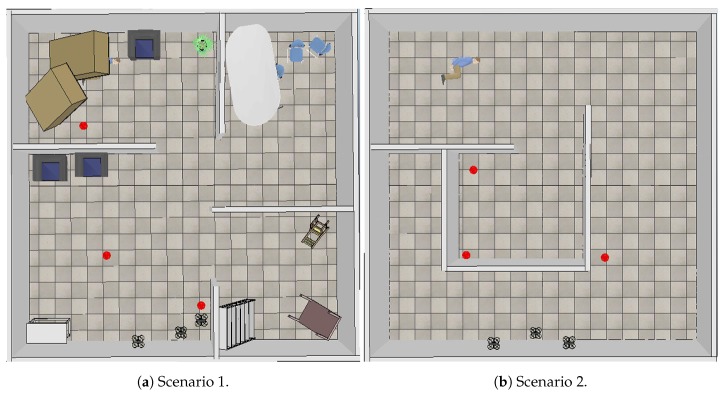
V-REP simulation scenarios.

Figure 8 shows the simulation scenarios. Both were 10 × 10 m locations. Scenario 1 (Figure 8a) presents a place characterized by rooms with furniture that were knocked down, like an earthquake scene, while Scenario 2 (Figure 8b) is a place with passages; red dots represent fire spots.

**Figure 9:**
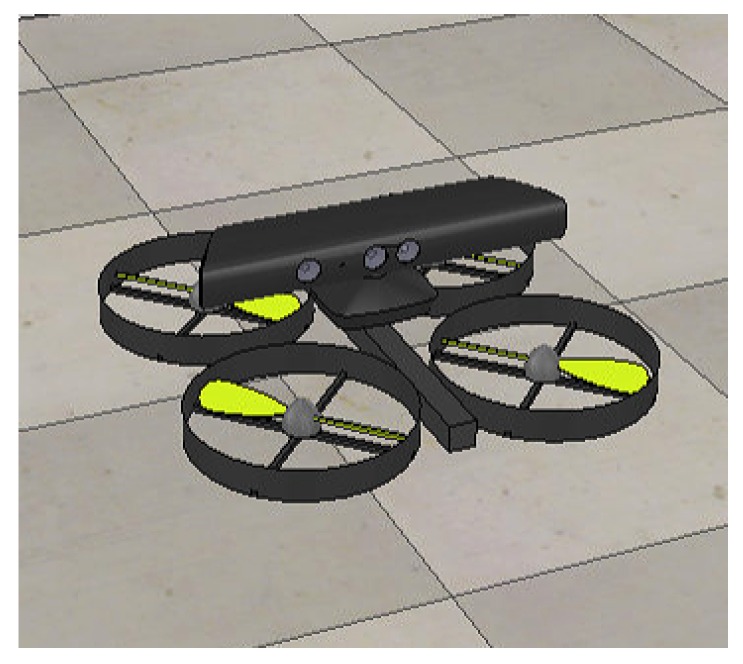
UAV in simulation.

To perform exploration, the simulations made use of two and three similar UAVs. Figure 9 shows a used UAV. The UAV was equipped with an RGB-D camera, a thermal sensor, and a laser sensor. The laser sensor took a 0.5 cm radio to the honeycomb, so distance from a hexagon center to another was 1 m. For each scenario and each configuration (two or three UAVs), simulations were performed with the FIFO and Euclidean Distance algorithms.

## 4. Results

**Figure 10:**
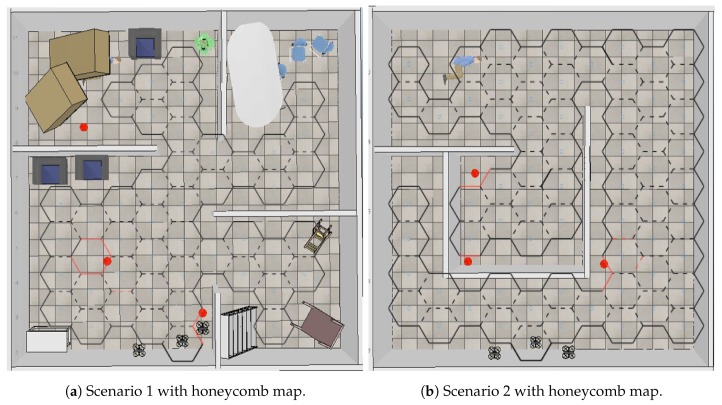
Scenarios merged with honeycomb map.

Figure 10 shows scenarios merged with the honeycomb-map build. After the simulations were performed, it was possible to verify the displacements of each UAV within the generated map, as well as the order of honeycomb exploration by each UAV.

### 4.1. Scenarios and Honeycomb-Map Generation

**Figure 11:**
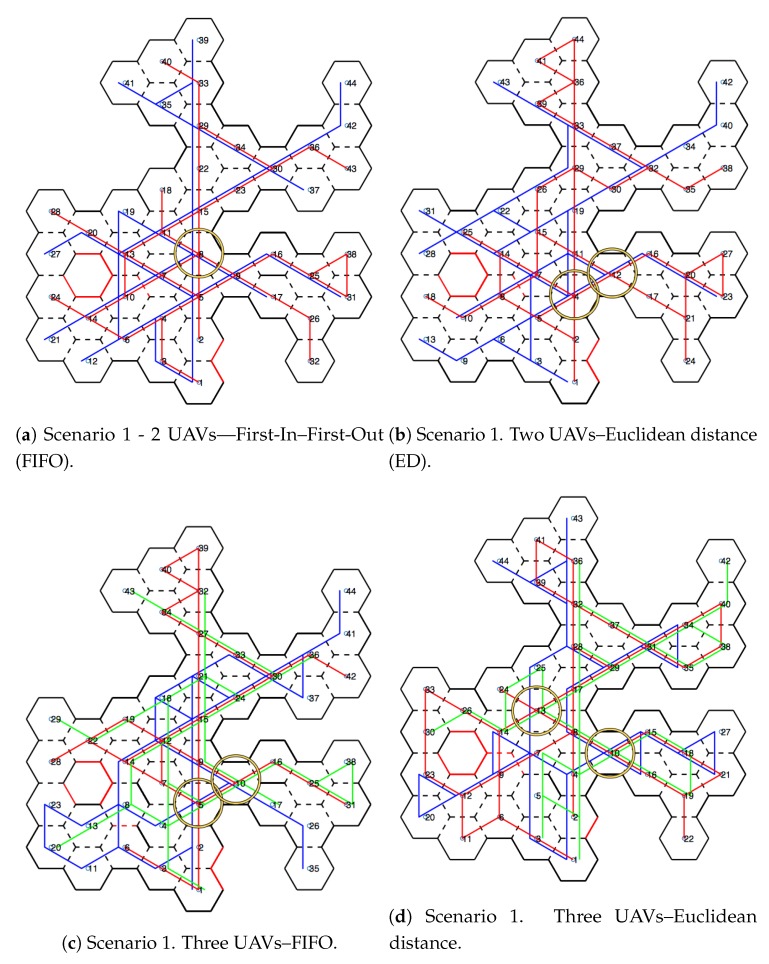
Displacement—Scenario 1.

**Figure 12:**
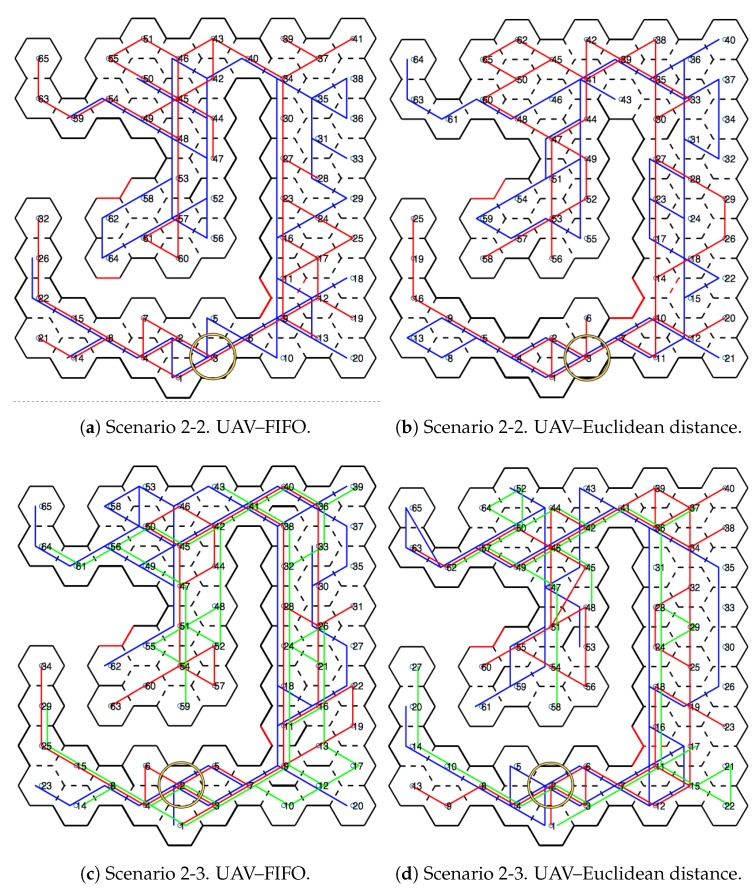
Displacement—Scenario 2.

**Figure 13:**
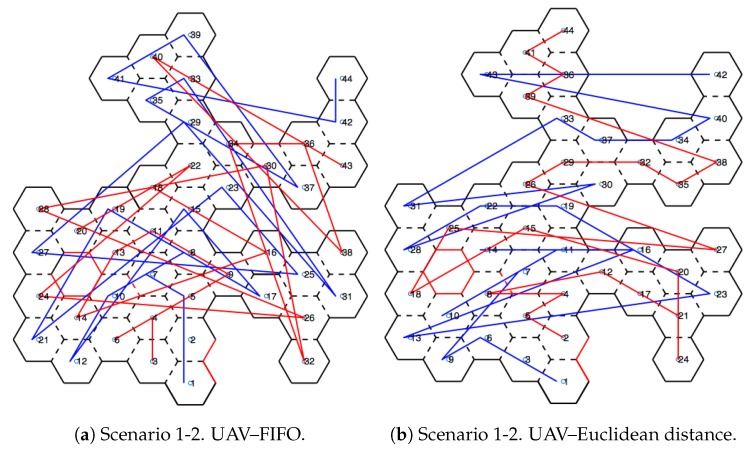

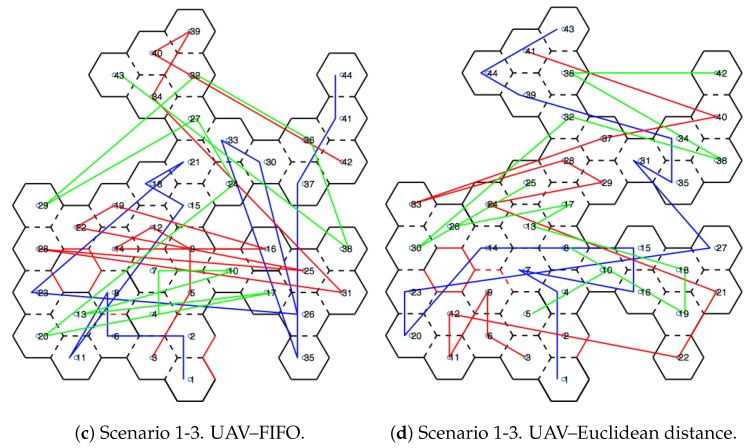
Exploration order—Scenario 1.

**Figure 14:**
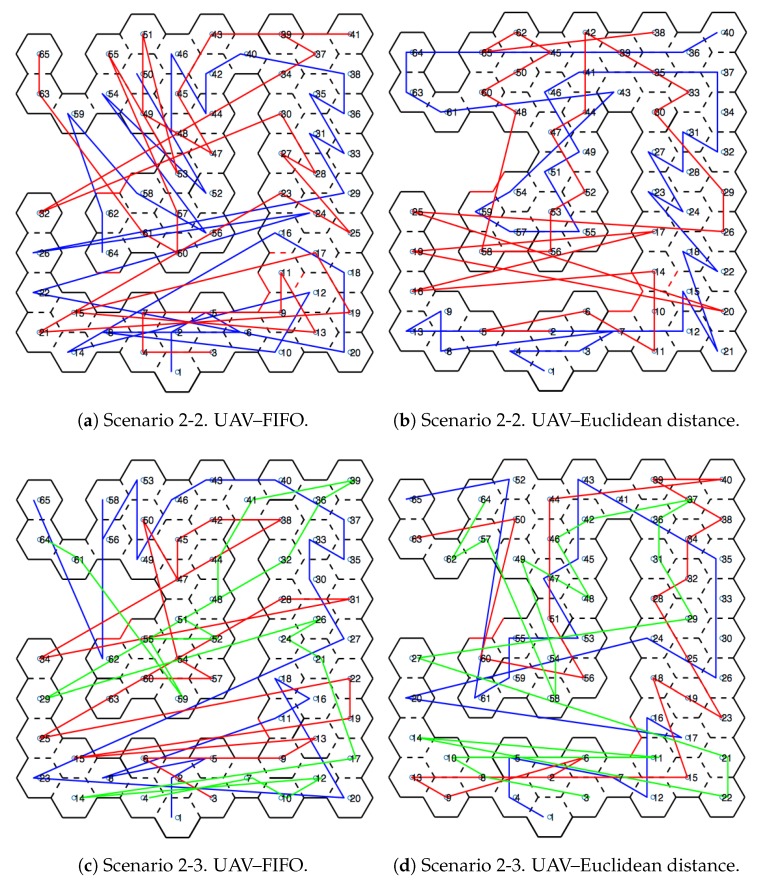
Exploration order—Scenario 2.

Figure 11 and Figure 12 show the movements made by the UAVs in the simulations of Scenarios 1 and 2, respectively. Figure 13 and Figure 14 present the exploration order of each UAV with the respective hexagon-definition algorithm to be explored, FIFO and Euclidean distance. The blue line correspond to UAV 1, the red is UAV 2, and the green is UAV 3 (when the simulation had three UAVs).

The yellow circle identifies the highest-traffic hexagon. Hexagon traffic means how many times a UAV went through the hexagon. Table 1 shows the max traffic number in the simulations. Considering the *ids* of Table 1, and relating them in Figure 11 and Figure 12, these most accessed hexagons were located in places characterized as doors or passageways.

In the simulations, the movements of each UAV were recorded. Displacement means that a UAV moved from a hexagon to an adjacent one. Table 2 shows the displacement number and average per UAV in each simulation in Scenario 1. Table 3 shows the same for Scenario 2. Table 4 bring the exploration time. Table 5 and Table 6 details data from both exploration order and displacement.

### 4.2. Cube View and Temperature Caption

**Figure 15:**
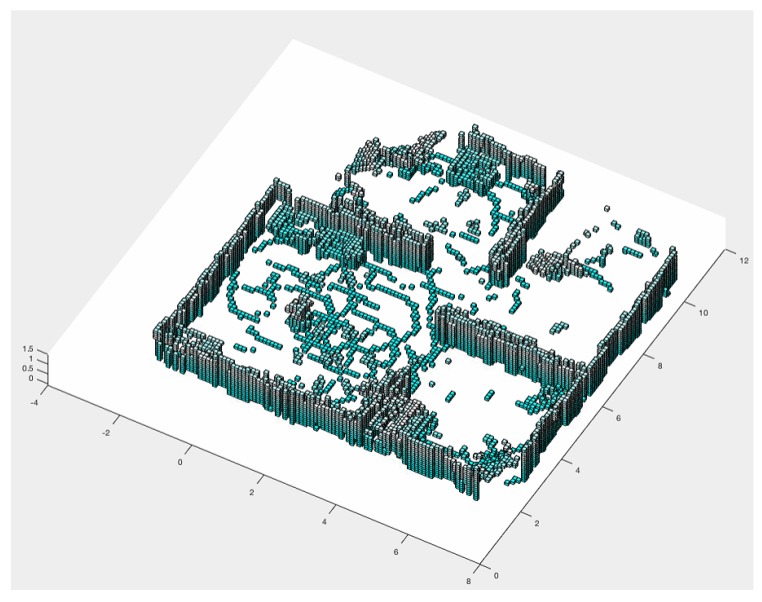
Three-dimensional cube view.

**Figure 16:**
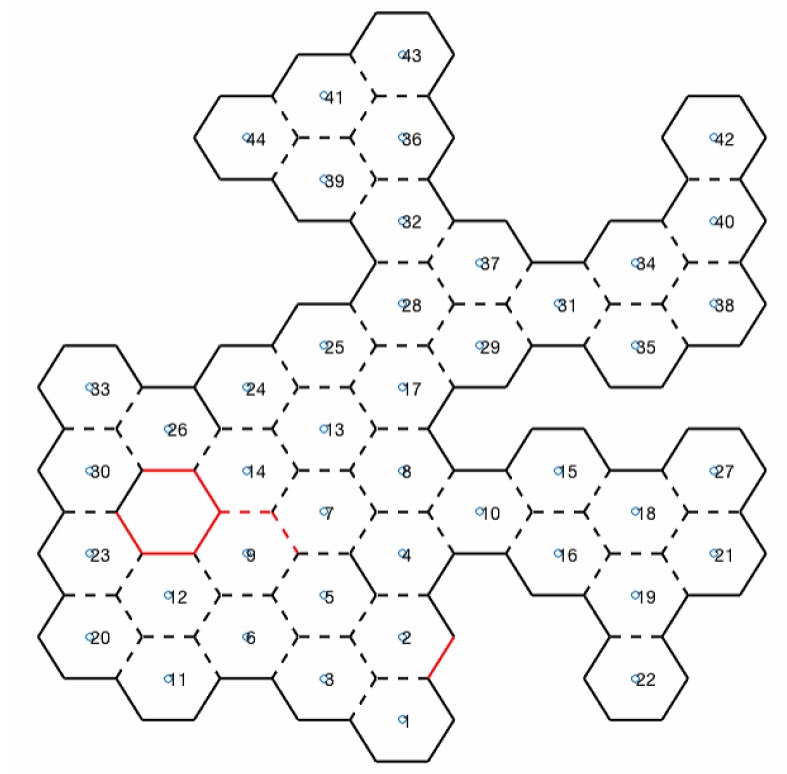
Honeycomb-map simulation—Scenario 1, three UAVs, Euclidean distance algorithm.

In addition to performing the mapping honeycomb in a hexagonal shape, further information can be generated for rescue teams. With the RGB-D sensor, a cube view can be generated. Figure 15 shows the Scenario 1 cube projection (Figure 8a), while Figure 16 shows the honeycomb map.

**Figure 17:**
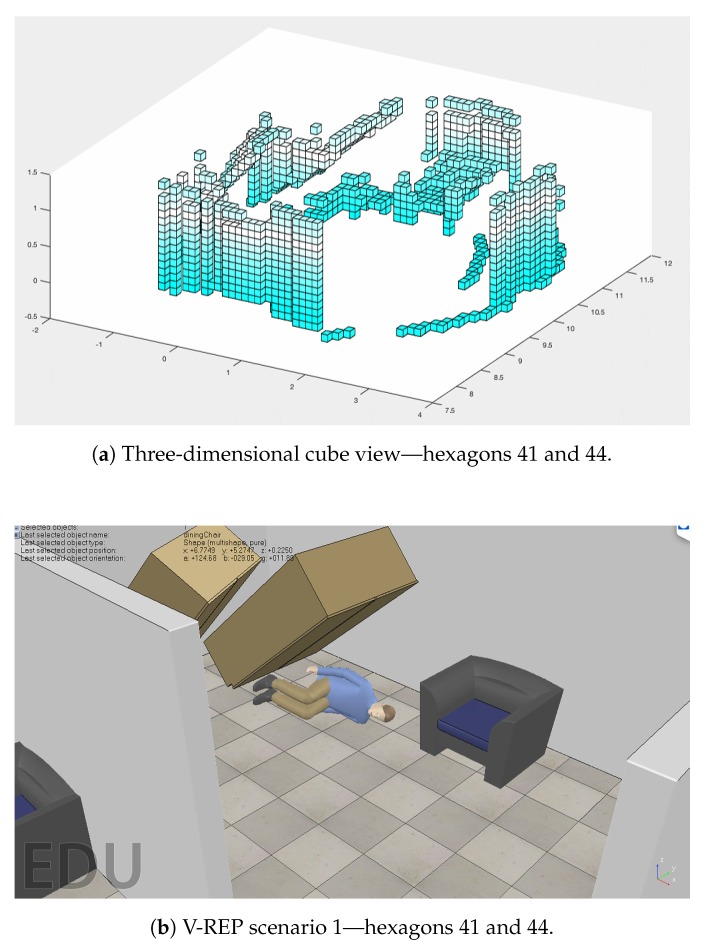
Clipping of hexagons 41 and 44 of Figure 16.

A cutout of 41 and 44 hexagons from the generated map of Figure 16, and the location of these hexagons in the simulator, are shown in Figure 17. In Section 3.1.7, the TransformRGB-D Algorithm 1 shows how RGB-D points are converted into 3D cubes.

**Figure 18:**
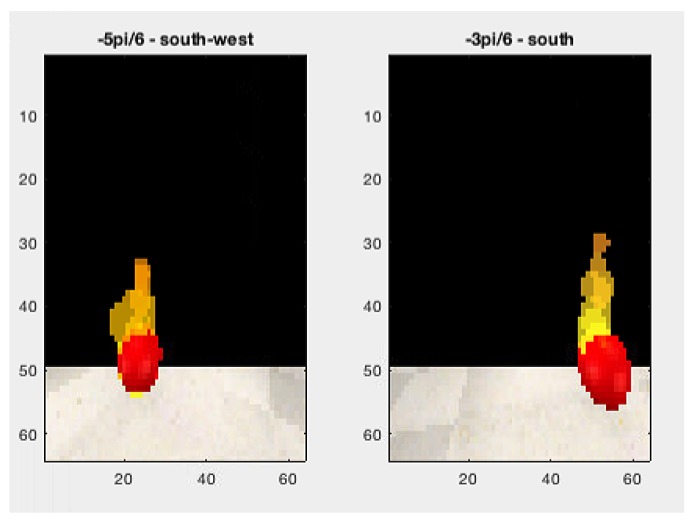
Fire-spot—hexagon 14 of Scenario 1.

In Figure 16, red lines (continuous or dashed) indicate the temperature reading above a reference value. Then, a fire-spot photo was recorded. Figure 18 shows the caption of hexagon 14 in Scenario 1.

## 5. Discussion

Topological mapping reduces information processing compared to metric mappings. The graph structure allows the execution of generic algorithms, such as the Dijkstra algorithm, used in trajectory planning. The data presented in Section 4 show the behavioral differences in the simulations considering number of UAVs, and algorithms in the definition of places to be explored, besides environment characteristics. By comparing the simulations, we verified that traffic in the hexagons was reduced when there was an algorithm change (FIFO for Euclidean Distance), as can be seen in Table 1. When comparing the change in UAV number, there was a slight increase in the maximum traffic value and exploration time, as can be seen in Table 4. When Scenario 2 is analyzed, the variation in the number of UAVs from two to three, in both FIFO and Euclidean Distance algorithms, reduces the exploration time. Already in Scenario 1 the opposite occurs. This happens due to the characteristics of the scenarios, where Scenario 2 has wide passages and more space for maneuvers, while Scenario 1 is composed of rooms and narrow doors, which influences the processing to avoid collisions in this points that were bottlenecks on the map. To decrease these values, an algorithm that considers not only Euclidean distance, but also the arrangement of UAVs and hexagons as a whole, should be evaluated.

On UAV displacement in the simulated scenario, Table 2 and Table 3 exhibited a strong reduction in UAV movement when increasing the number of UAVs and changing the algorithm of choosing hexagons to explore. For Scenario 1, the change in the number of UAVs in the FIFO algorithm showed 31.63% reduction in average displacement per UAV. For the Euclidean distance algorithm, the reduction was 21.21%. By changing the simulation with two UAVs to three, and the FIFO algorithm for the Euclidean distance algorithm, reducing displacement in the scenario reached 42.52%; the same analysis for Scenario 2 showed a displacement decrease of 37.7%. This saves both energy and exploration time.

## 6. Conclusions

This work presented an environment mapping method inspired by how bees build their hives. Since only one bee constructs and occupies the space of a honeycomb, a topological map was constructed so that UAVs involved in the mapping process behaved similarly to bees. The definition of which honeycomb the UAV should map depends on a metric. The performed simulations considered two metrics to define which honeycomb should be mapped, FIFO and Euclidean Distance. In addition, simulations were performed by changing the number of UAVs. This demonstrated that setting the exploration order has direct impact on the number of offsets and a UAV in the environment, considering its position on the map. This can result in saving both energy and exploration time. Generating RGB-D and thermal-reading information enables rescuers to be prepared for obstacles and dropped objects, but also life-threatening elements such as high temperatures.

### Future Work

Improvements in the definition of the spaces to be explored can be made, with metrics that consider not only distance from the initial hexagon (Euclidean distance), but also UAV location and environmental characteristics. In addition, in identifying points that may endanger the life of the rescue team, the use of gas or other toxic-element sensors may be applied. There is still the challenge of gathering this information and processing it with the use of game theory and machine learning. So far, each UAV works independently; however, it is not identified when a failure occurs with another one. A way of detecting failures and generating contingency plans needs to be implemented in future work. In this work, the representation of the hexagons is made in a projection of the *x* and *y* axes. In future work, the *z* axis will be added, so that this representation has several layers.

## Figures and Tables

**Table 1:** Hexagon traffic.

Simulation	Scenario 1		Scenario 2	
	Id Hexagon	Traffic Number	Id Hexagon	Traffic Number
Two UAVs–FIFO	8	17	3	19
Two UAVs–Euclidean distance	4 and 12	8	3	13
Three UAVs–FIFO	5 and 10	15	2	21
Three UAVs–Euclidean distance	10 and 13	9	12	14

**Table 2:** Displacement number—Scenario 1.

Simulation	FIFO	Average/UAV	Euclidean Distance	Average/UAV
Two UAVs	196	98	143	71.5
Three UAVs	203	67	169	56.33
Variation	-	31.63%	-	21.21%

**Table 3:** Displacement number—-Scenario 2.

Simulation	FIFO	Average/UAV	Euclidean Distance	Average/UAV
Two UAVs	290	145	206	103
Three UAVs	324	108	271	90.33
Variation	-	25.51%	-	12.29%

**Table 4:** Exploration time.

Simulation	Scenario 1	Scenario 2
Two UAVs–FIFO	2:30:32	3:00:17
Two UAVs–Euclidean distance	2:24:56	2:27:58
Three UAVs–FIFO	3:44:25	2:08:09
Three UAVs–Euclidean distance	3:54:17	1:56:08

**Table 5:** Exploration order.

Scenarios	UAV Number	Exploration Order	
Scenario 1	**Three UAV - Euclidean Distance**		
	UAV 1	1 2 4 7 16 15 14 20 23 27 31 35 34 39 44 43	
	UAV 2	3 6 9 11 12 22 21 24 29 28 33 37 40 41	
	UAV 3	5 10 8 19 18 13 17 26 25 30 32 38 36 42	
	**Two UAV - Euclidean Distance**		
	UAV 1	1 3 6 9 7 10 11 14 16 13 23 19 22 28 30 31 33 37 34 40 43 42	
	UAV 2	2 5 4 8 12 17 21 24 20 15 18 25 27 26 29 32 35 38 39 36 41 44	
	**Three UAV - FIFO**		
	UAV 1	1 2 6 8 11 15 18 21 23 26 30 33 35 37 41 44	
	UAV 2	3 5 9 12 14 16 19 22 25 28 31 34 39 40 42	
	UAV 3	4 7 10 13 17 20 24 27 29 32 36 38 43	
	**Two UAV - FIF**O		
	UAV 1	1 2 5 7 8 10 12 15 17 19 21 23 25 27 29 31 33 35 37 39 41 42 44	
	UAV 2	3 4 6 9 11 13 14 16 18 20 22 24 26 28 30 32 34 36 38 40 43	
Scenario 2	**Three UAV - Euclidean Distance**		
	UAV 1	1 4 5 7 12 16 17 20 24 26 30 33 35 41 43 45 47 53 55 59 61 52 65	
	UAV 2	2 6 9 13 15 18 19 23 25 28 32 34 38 39 40 44 51 56 60 50 63	
	UAV 3	3 8 10 11 14 22 21 27 29 31 36 37 42 46 48 49 54 58 57 62 64	
	**Two UAV - Euclidean Distance**		
	UAV 1	1 4 3 7 8 9 13 12 15 21 18 22 23 24 27 28 31 32 34 37 41 46 49 51 55 57 59 54 43 61 63 64 36 40	
	UAV 2	2 5 6 11 10 14 16 17 19 20 25 26 29 30 33 35 39 42 44 47 52 53 56 58 48 60 50 45 62 65 38	
	**Three UAV - FIFO**		
	UAV 1	1 2 5 8 11 16 18 20 23 27 30 33 35 37 40 43 46 49 53 56 58 62 65	
	UAV 2	3 6 9 13 15 19 22 25 28 31 34 38 42 45 47 50 54 57 60 63	
	UAV 3	4 7 10 12 14 17 21 24 26 29 32 36 39 41 44 48 51 52 55 59 61 64	
	**Two UAV - FIFO**		
	UAV 1	1 2 5 6 8 10 12 14 16 18 20 22 24 26 29 31 33 35 36 38 40 42 44 46 48 50 52 54 56 58 59 62 64	
	UAV 2	3 4 7 9 11 13 15 17 19 21 23 25 27 28 30 32 34 37 39 41 43 45 47 49 51 53 55 57 60 61 63 65	

**Table 6:** Displacement order.

Scenarios	UAV Number	Exploration Order	
Scenario 1	**Three UAV - Euclidean Distance**	
	UAV 1	1 2 4 7 4 10 16 15 10 4 7 14 9 12 20 23 12 9 7 4 10 15 18 21 27 18 15 10 8 17 29 31 35 34 31 29 28 32 39 44 39 36 43 36 32 28 25 13 7 5 3 1
	UAV 2	1 3 6 9 6 11 12 9 7 8 10 16 19 22 19 21 18 15 10 8 13 24 13 17 29 28 17 13 14 9 12 23 30 33 26 14 13 17 28 32 37 31 35 38 40 34 31 29 28 32 36 41 39 32 28 17 8 4 2
	UAV 3	1 2 5 2 4 10 8 10 16 19 18 15 10 8 13 17 13 14 26 14 24 25 13 14 26 30 26 14 13 17 28 32 28 29 31 35 38 34 31 37 32 36 32 37 31 34 40 42 40 34 31 29 17 8 4 7 5 3
	**Two UAV - Euclidean Distance**	
	UAV 1	1 3 6 9 6 5 7 8 10 8 7 11 7 14 7 4 12 16 12 4 5 6 9 13 9 6 5 4 12 16 20 23 20 16 12 11 19 15 22 25 28 25 14 15 19 30 19 15 14 25 31 25 22 26 29 33 37 32 34 40 34 32 37 33 39 43 39 33 37 32 34 40 42 40 34 32 30 19 11 4 5 3 1
	UAV 2	1 2 5 4 5 8 5 4 12 17 21 24 21 20 16 12 11 15 7 8 10 18 10 8 14 25 14 7 4 12 16 20 23 27 20 16 12 11 15 26 29 30 32 35 38 35 32 37 33 39 36 41 44 36 33 29 19 11 4 2
	**Three UAV - FIFO**	
	UAV 1	1 2 5 2 3 6 8 6 11 6 8 4 5 9 15 18 21 15 12 14 8 6 11 20 23 13 8 4 5 10 17 26 17 10 9 15 24 30 33 21 15 9 10 17 26 35 26 17 10 9 12 18 21 24 30 37 36 41 44 41 36 30 24 15 9 5 4
	UAV 2	1 3 6 3 1 2 5 9 12 14 7 5 10 16 10 9 12 19 22 14 7 5 10 16 25 16 10 5 7 14 22 28 22 14 7 5 10 16 25 31 25 16 10 5 7 12 15 21 27 34 32 39 40 32 27 33 30 36 42 36 30 24 15 9 5 2
	UAV 3	1 3 4 7 5 10 5 4 8 13 8 4 5 10 17 10 5 4 8 13 20 13 8 14 12 15 24 21 27 21 18 19 22 29 22 19 18 21 27 32 27 33 30 36 30 24 15 9 10 16 25 31 38 25 16 10 9 15 21 27 34 43 34 27 21 18 12 7 4 3
	**Two UAV - FIF**O	
	UAV 1	1 2 5 7 8 7 10 6 12 6 4 5 8 15 8 9 17 9 8 11 19 13 10 14 21 14 10 7 8 15 23 15 8 9 16 25 16 9 5 7 13 20 27 20 13 11 15 22 29 22 15 8 9 16 25 31 25 16 9 8 15 22 29 33 35 29 34 30 37 30 34 29 33 39 33 35 41 35 29 34 30 36 42 44 42 36 30 23 15 8 5 4 3 1
	UAV 2	1 3 4 6 4 5 9 8 11 13 10 14 6 4 5 9 16 9 8 11 18 11 13 20 13 11 15 22 15 8 7 10 14 24 14 6 4 5 9 17 26 17 9 5 7 13 20 28 20 13 11 15 23 30 23 15 8 9 17 26 32 26 17 9 8 15 22 29 34 30 36 30 23 15 8 9 16 25 31 38 25 16 9 8 15 22 29 33 40 33 29 34 30 36 43 36 30 23 15 8 5 2
Scenario 2	**Three UAV - Euclidean Distance**	
	UAV 1	1 4 5 2 3 7 12 11 16 17 11 7 3 2 4 8 10 14 20 14 10 8 4 2 3 7 12 15 17 16 18 24 18 19 26 30 33 35 34 36 41 43 42 45 47 46 47 48 53 48 47 51 55 59 61 59 54 51 47 46 44 52 44 50 57 62 65 63 62 57 49 46 42 41 36 31 28 24 18 16 11 7 6 2 1
	UAV 2	1 2 3 6 2 4 8 9 13 9 8 4 2 6 7 11 15 11 16 18 19 23 19 25 24 28 32 34 38 34 36 39 37 40 37 36 41 42 44 46 45 51 48 51 55 51 48 53 56 54 55 60 55 51 47 46 50 57 49 57 50 57 50 57 49 57 62 63 62 57 49 46 42 41 39 37 34 32 29 25 19 17 15 12 7 6 2
	UAV 3	1 3 7 3 2 4 8 10 8 4 2 3 7 11 7 3 2 4 8 10 14 10 8 4 2 3 7 11 15 22 21 15 11 17 11 7 3 2 4 8 10 14 20 27 20 14 10 8 4 2 3 7 11 16 18 24 29 28 31 36 37 36 41 42 46 45 48 45 46 49 47 51 54 58 54 51 47 49 57 62 57 50 57 50 52 64 50 44 42 41 36 31 28 24 18 16 11 7 3
	**Two UAV - Euclidean Distance**	
	UAV 1	1 4 1 3 7 3 2 4 5 8 5 9 13 8 5 4 2 3 7 10 12 15 12 21 12 15 18 15 22 18 17 23 24 23 27 28 31 32 34 37 33 35 39 41 46 41 44 49 51 49 52 55 53 57 59 54 51 47 44 41 43 41 46 48 60 61 63 64 63 61 60 48 46 41 39 35 36 40 36 33 31 28 24 18 15 12 11 7 3 1
	UAV 2	1 2 4 5 4 2 3 6 3 7 11 10 14 10 7 3 1 4 5 9 16 9 5 4 2 3 7 10 14 17 14 10 7 3 2 4 5 9 16 19 16 9 5 4 2 3 7 10 12 20 12 10 7 3 2 4 5 9 16 19 25 19 16 9 5 4 2 3 7 10 14 18 26 29 28 27 30 33 35 39 42 41 44 47 49 52 53 56 53 57 58 57 53 51 47 48 60 50 45 62 65 50 45 41 39 38 35 30 27 23 17 14 10 7 3 2
	**Three UAV - FIFO**	
	UAV 1	1 2 5 2 4 8 4 2 3 7 9 11 16 18 11 9 12 20 12 9 7 3 2 4 8 14 23 14 8 4 2 3 7 9 11 16 22 27 26 30 33 30 35 37 36 40 36 39 36 38 41 40 41 38 41 40 41 40 41 38 43 46 45 49 45 46 53 50 56 50 53 58 50 45 47 51 55 62 55 51 47 49 56 61 56 49 56 61 64 65 64 61 56 49 45 42 41 38 32 28 24 18 11 9 7 5 2 1
	UAV 2	1 3 2 3 2 3 2 4 6 2 3 7 9 13 9 7 3 2 4 8 15 8 4 2 3 7 9 13 19 22 16 11 9 7 3 2 4 8 15 25 15 8 4 2 3 7 9 11 18 24 28 26 31 26 21 16 11 9 7 3 2 4 8 15 25 29 34 29 25 15 8 4 2 3 7 9 11 18 24 28 32 38 41 42 45 47 44 42 46 50 45 47 51 54 52 57 54 60 63 60 54 51 47 44 42 41 40 36 33 30 26 21 16 11 9 7 5 2
	UAV 3	1 3 7 3 2 4 8 10 8 4 2 3 7 11 7 3 2 4 8 10 14 10 8 4 2 3 7 11 15 22 21 15 11 17 11 7 3 2 4 8 10 14 20 27 20 14 10 8 4 2 3 7 11 16 18 24 29 28 31 36 37 36 41 42 46 45 48 45 46 49 47 51 54 58 54 51 47 49 57 62 57 50 57 50 52 64 50 44 42 41 36 31 28 24 18 16 11 7 3
	**Two UAV - FIFO**	
	UAV 1	1 2 3 5 6 3 2 4 8 4 2 3 6 10 9 12 9 6 3 2 4 8 14 8 4 2 3 6 9 11 16 11 12 18 12 13 20 13 9 6 3 2 4 8 15 22 15 8 4 2 3 6 9 11 16 24 16 11 9 6 3 2 4 8 15 22 26 22 15 8 4 2 3 6 9 11 16 24 29 28 31 33 31 35 36 38 35 34 40 42 44 42 46 45 48 45 50 45 44 47 52 47 48 49 54 49 48 47 52 56 57 53 58 53 48 49 54 59 54 49 48 53 58 62 64 61 57 52 47 44 42 40 34 30 27 23 16 11 9 6 3 1
	UAV 2	1 3 1 4 7 2 3 6 9 11 9 13 9 6 3 2 4 8 15 8 4 2 3 6 9 11 17 12 19 12 9 6 3 2 4 8 14 21 14 8 4 2 3 6 9 11 16 23 16 17 25 24 23 27 28 27 30 27 23 16 11 9 6 3 2 4 8 15 22 26 32 26 22 15 8 4 2 3 6 9 11 16 23 27 30 34 37 39 37 41 37 34 40 43 42 45 44 47 44 45 49 45 46 51 46 45 48 53 48 45 46 51 55 50 45 48 53 48 53 57 60 61 57 53 48 49 54 59 63 65 63 59 54 49 45 46 43 40 34 30 27 23 16 11 9 6 3 2

## Abbreviations

The following abbreviations are used in this manuscript:

AD	Adjacent Degree
ED	Euclidean Distance
EKF	Extended Kalman Filter
FIFO	First-In–First-Out
GPS	Global Positioning Systems
IMU	Inertial Measurement Unit
IR	Infrared
JCBB	Join-compatibility Branch and Bound
PTAM	Parallel Tracking and Mapping
PTAMM	Parallel Tracking and Multiple Mapping
RGB-D	Red, Green, Blue and depth
SLAM	Simultaneous Localization and Mapping
SPF	Stigmergic Potential Field
UAV	Unmanned Aerial Vehicle
UGV	Unmanned Ground Vehicle
VSLAM	Visual SLAM
